# Clinical and Real-World Effectiveness of Mogamulizumab: A Narrative Review

**DOI:** 10.3390/ijms25042203

**Published:** 2024-02-12

**Authors:** Montserrat Fernández-Guarino, Pablo Ortiz, Fernando Gallardo, Mar Llamas-Velasco

**Affiliations:** 1 Dermatology Department, Hospital Universitario Ramón y Cajal, Instituto de Investigación Sanitaria Ramón y Cajal (Irycis), 28034 Madrid, Spain; montserrat.fernandezg@salud.madrid.org; 2 Dermatology Department, Hospital 12 de Octubre, 28041 Madrid, Spain; plortizr@gmail.com; 3 Dermatology Department, Hospital del Mar, 08003 Barcelona, Spain; 2fgallardo@psmar.cat; 4 Dermatology Department, Hospital Universitario de la Princesa, Fundación de Investigación Biomédica de la Princesa, 28006 Madrid, Spain

**Keywords:** cutaneous T-cell lymphoma, effectiveness, mogamulizumab, peripheral T-cell lymphoma

## Abstract

Mogamulizumab (MOG) is an antibody targeting the CCR4 receptor, authorized for relapsed or refractory peripheral T-cell (PTCL) and cutaneous T-cell lymphomas (CTCL). Its adoption in guidelines and endorsement by FDA and EMA established it as a systemic treatment, especially for advanced disease stages due to its comparatively lower toxicity. Clinical trials and real-world evidence have underscored its efficacy in advanced CTCLs, including mycosis fungoides and Sézary syndrome; PTCLs; and adult T-cell leukemia/lymphoma (ATLL), showcasing positive outcomes. Notably, the drug has demonstrated significant response rates, disease stability, and extended periods of progression-free survival, suggesting its applicability in cases with multiple treatment lines. Its safety profile is generally manageable, with adverse events (AEs) primarily related to the skin, infusion-related reactions, drug eruptions, autoimmune diseases, and skin disorders. The latter seem to appear as CCR4 can promote the skin-specific homing of lymphocytes, and MOG is directed against this receptor. While combination with immunostimulatory agents like interferon alpha and interleukin 12 has shown promising results, caution is urged when combining with PD1 inhibitors due to the heightened risk of immune-mediated AEs. The introduction of MOG as a systemic treatment implies a significant advancement in managing these diseases, supported by its favorable safety profile and complementary mechanisms.

## 1. Introduction

Mogamulizumab (MOG) is a humanized defucosylated monoclonal antibody that targets the transmembrane chemokine receptor 4 (CCR4). While CCR4 expression is typically confined to regulatory T cells (Treg) and type 2 T helper (Th2) cells in normal environments, this membranous receptor is found to be overexpressed in certain T-cell malignancies, notably in advanced stages of mycosis fungoides (MF) and Sezary syndrome (SS).

Mycosis fungoides and Sezary syndrome are T-cell lymphomas, and they have been recently reviewed [1,2,3]. The binding of MOG to CCR4-expressing cells has been observed to potentiate antibody-dependent cellular cytotoxicity (ADCC), and subsequently leads to the depletion of the target cells. However, resistance to treatment might arise due to novel CCR4 mutations that target the N-terminal and transmembrane domains, contributing to disease progression [4].

MOG has been approved by the Food and Drug Administration (FDA) for the treatment of relapsed and refractory peripheral T-cell lymphoma (PTCL) and cutaneous T-cell lymphoma (CTCL). Its use has been extended to refractory or relapsed cases of MF and SS following the failure of at least one systemic therapy, as evidenced by the results of the MAVORIC trial. Based on data from six clinical trials, a pharmacokinetic (PK) model analyzing the drug levels of MOG revealed that certain factors such as albumin levels, aspartate aminotransferase, mild-to-moderate hepatic impairment, and sex are statistically significant predictors of its clearance [5].

Treatment with MOG leads to a significant and enduring decrease in peripheral blood CD4+, CD25+, FoxP3+ natural Treg cells. Notably, it was first recognized as a treatment option for relapsed or refractory CCR4+ adult T-cell leukemia/lymphoma (ATLL) in Japan back in 2012. Subsequently, its efficacy was acknowledged for relapsed refractory PTCL and CTCL in 2014, and it gained approval from the FDA in August 2018 for treating relapsed/refractory CTCL in adult patients who had undergone at least one prior systemic therapy. The European Medicines Agency (EMA) also authorized the use of the drug throughout the European Union (EU) on 22 November 2018.

## 2. Mechanism of Action

MOG is a humanized monoclonal antibody that specifically recognizes the N-terminal region of human CCR4 [6], a seven-transmembrane G-protein-coupled receptor. This receptor is widely expressed in a variety of cell types, including Th2, Treg, memory T cells, cutaneous lymphocyte-associated antigen (CLA)-positive T cells, monocytes, and platelets. Among these cell types, notably, Th2 and Treg cells exhibit a selective and high expression of CCR4. While the exact role of CCR4 in ATLL cells remains unclear, approximately 90% of ATLL patients express a robust CCR4 expression [7]. Additionally, some patients with PTCL, distinct from ATLL or CTCL, exhibit varying levels of CCR4 expression. Consequently, CCR4 emerges as a promising therapeutic target, particularly in T-cell lymphomas like ATLL.

**Figure 1:**
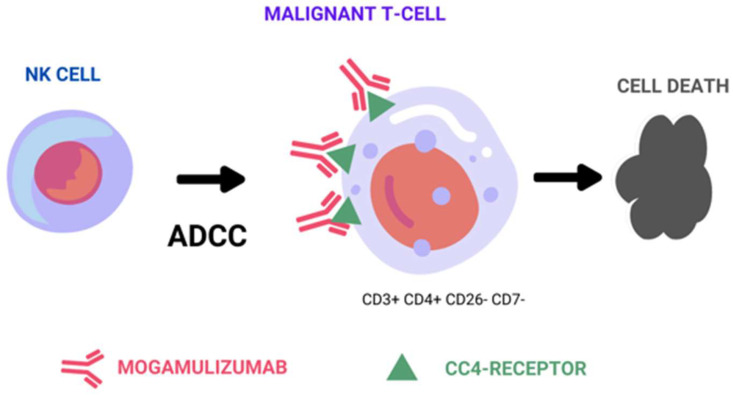
Representative figure of mogamulizumab’s main mechanism of action by antibody-dependent cell-mediated cytotoxicity.

Therapeutic monoclonal antibodies achieve their effectiveness against tumor cells through three distinct mechanisms: the above-mentioned ADCC mechanism, complement-dependent cytotoxicity (CDC), and direct cell death (Figure 1). Among these mechanisms, ADCC is considered a representative in vivo antitumor mechanism of therapeutic antibodies, such as rituximab, trastuzumab, and alemtuzumab [8,9,10]. ADCC relies on the presence of oligosaccharides in the Fc region of the antibody, and is sensitive to changes in the sugar chain structure [11,12]. Shinkawa et al. have reported that a fucose-containing sugar chain in the Fc region plays a pivotal role in influencing ADCC activity, demonstrating that defucosylated IgG1 enhances ADCC activity more than 50-fold [13]. Subsequently, Iida et al. reported that defucosylation of the Fc region sugar chain increases the binding affinity to the Fc-gamma receptor on effector cells, resulting in heightened ADCC activity [14,15].

As a result of these discoveries, researchers have developed MOG (KW-0761), a defucosylated humanized monoclonal antibody that targets CCR4 [16]. Consequently, MOG primarily operates by potently triggering ADCC against CCR4-positive tumor cells, with no observed evidence of CDC or direct cell death. In addition to depleting tumor cells, it also effectively engages Tregs expressing CCR4 on their cell surface through ADCC. This dual-action mechanism has the potential to enhance the immune response of cytotoxic T cells against tumor cells, thereby augmenting the efficacy of mogamulizumab in combating cancer [17].

Regarding the PK profile of MOG, mean values of trough (Ctrough) and maximum (Cmax) plasma concentration were established in a phase I and II studies at 33.6 μg/mL and 42.9 μg/mL, respectively, at a dose of 1.0 mg/kg, with a mean elimination half-life of 17.5 days [18,19].

## 3. Mogamulizumab in Clinical Guidelines

MOG was approved by the EMA in 2018 for the treatment of patients with CTCL who have received at least one line of systemic therapy.

Since then, MOG has been featured in various updated guidelines. However, the British guidelines published in 2018 did not include it [20]. All guidelines focused on CTCL treatment commonly divide therapies into those targeting early or localized stages of the disease (IA–IIA) and systemic therapies for advanced or extensive stages (IIB or higher). MOG falls into the latter category as a new therapeutic alternative.

In the American National Comprehensive Cancer Network (NCCN) guidelines [21], MOG is listed as a category A systemic treatment. Category A drugs are considered preferable due to their lower toxicity. Since there is no curative alternative for CTCL, the guidelines suggest selecting treatment regimens based on a progression from lower to higher toxicity as the response diminishes. Other recommended treatments in category A include classic options like methotrexate, bexarotene, interferon alpha-2b, or interferon-gamma-1b, as well as newer and targeted therapies such as brentuximab, vorinostat, and romidepsin. MOG is not recommended for advanced MF transformation into large cells due to the lack of supporting studies. The guidelines also recommend selecting treatments that allow for low-toxicity maintenance regimens to avoid switching therapies, as in the case of MOG. The superior results of the MAVORIC study in advanced stages are highlighted when choosing between treatment options, particularly as MOG demonstrated better response rates in patients with stages III (23%) or IV (36%), and those with peripheral blood involvement.

In 2020, the Japanese dermatology guidelines were published, in which a review of the management of primary cutaneous T-cell lymphomas was included [22]. These guidelines consider MOG as a treatment option for MF in stages IIB to IV following the failure of classic treatments, which include interferon or retinoids like acitretin or bexarotene. Therefore, they align with the American NCCN guidelines. Caution is advised when using MOG in patients undergoing allogeneic hematopoietic cell transplantation, as isolated cases of ATLL and CTCL have indicated an increased likelihood of post-transplant graft-versus-host disease (GVHD) development.

The 2021 Spanish “GELTAMO” guidelines, developed by a consensus of 12 experts, including hematologists, dermatologists, pathologists, and oncologists, positioned MOG in an enriching manner within therapeutic groups [23]. In these guidelines, MOG is included in the treatment of MF and SS as a second-line systemic treatment. The guidelines distinguish between different treatment groups, including immunomodulatory agents, monoclonal antibodies, chemotherapy, and histone deacetylase inhibitors (HDACi). They also discussed classic combinations of these with psoralen plus ultraviolet A (PUVA) phototherapy or extracorporeal photopheresis, though not with MOG. In the Spanish guidelines, MOG is indicated for patients with MF with spots or plaques at stage IB or higher as a second-line treatment after the failure of skin-directed treatment and/or classic treatments such as photopheresis, interferon, radiotherapy, retinoids, or methotrexate. An exception is stage IV, where it is recommended as a first-line treatment with other antibodies or chemotherapy or allogeneic transplantation.

These guidelines differentiate between advanced SS and advanced MF, placing MOG as a second-line treatment with other antibodies, allogeneic hematopoietic progenitor cell transplantation, or chemotherapy after the failure of classic treatments. Photopheresis, chlorambucil plus prednisone, retinoids plus photopheresis, electron beam therapy, or retinoids plus PUVA are considered classic treatments for SS. The guidelines also highlighted the higher incidence of GVHD in patients who received MOG before allogeneic transplantation in ATLL (81% versus 41%), thus recommending careful optimization of transplant preparation in SS patients and delaying it after MOG treatment.

The European Organization for Research and Treatment of Cancer (EORTC) recently updated its CTCL and SS treatment guidelines in 2023 [1]. These guidelines include not only MOG but also brentuximab as targeted treatment. Pegylated forms of interferon were updated following the discontinuation of the nonpegylated form, with topical mechlorethamine, which is not available in all countries, also being added. HDACis were not covered as they are not approved for CTCL in Europe. In localized forms of MF, skin-directed treatment remains the most recommended one according to these experts. In advanced stages, the guidelines emphasize the incurability of the disease and prioritize patient quality of life by seeking the fewest side effects. In the latest EORTC guidelines, MOG follows the EMA approval recommendations as a treatment for adult patients with MF or SS who have failed to at least one systemic therapy. These are recommendations either for patients in early stages or advanced stages (IIB or higher). Additionally, no specific preference order is established. EORTC guidelines mention a common yet peculiar situation in clinical practice, which involves the presentation of MF or SS in elderly patients and the need for special care considerations and treatment selection. In this case, MOG has not shown differences in results by age, but caution was advised as there were insufficient age-segmented studies to make recommendations.

**Figure 2:**
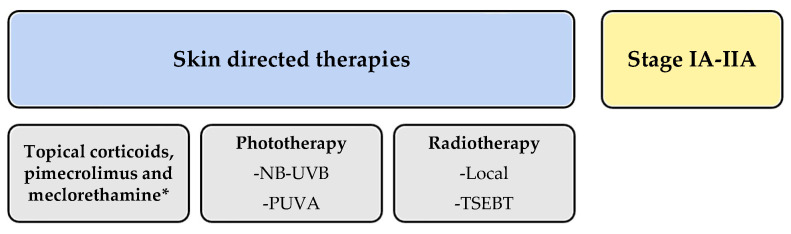

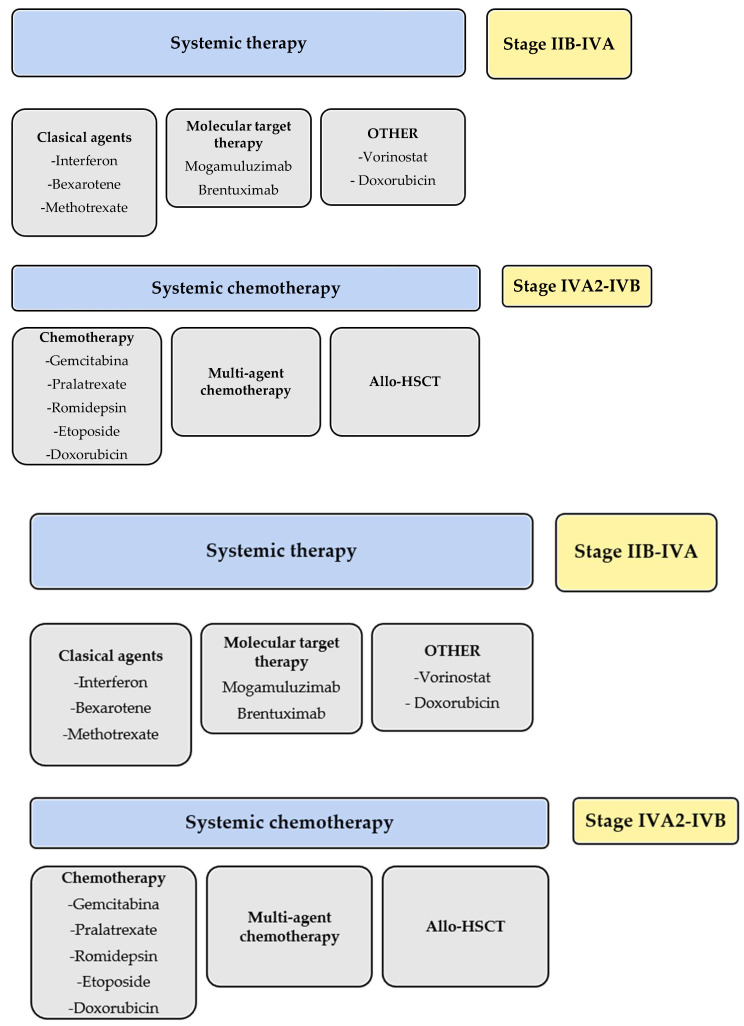
Schematic summary of the guidelines for treatment of mycosis fungoides and Sezary syndrome. * meclorethamine is not available in every country.

In summary, all guidelines positioned MOG as a targeted antibody treatment indicated after systemic treatment failure in different stages MF (IIB-IVA, Figure 2).

In addition to treatment guidelines, several expert-reviewed publications provided different perspectives. Sugaya et al. reviewed the classification and new target treatments for cutaneous lymphomas [24]. While it described all molecular mechanisms, it did not provide recommendations, making it an interesting read for in-depth understanding. On the other hand, Kamijo et al. [25] made a comprehensive review of current treatment guidelines, excluding the latest EORTC guidelines, recommending a logical treatment choice in advanced forms of MF or SS after failed classic treatments. Brentuximab was recommended due to CD30 positivity, and MOG for blood involvement.

## 4. Clinical Trials Involving Mogamulizumab

The phase I/II open-label multicenter study conducted by Duvic et al. evaluated MOG in 41 patients with MF/SS, showing no dose-limiting toxicity. In the phase II trial, 38 patients received MOG at a dose of 1 mg/kg weekly for four weeks, followed by maintenance of the same dose every two weeks until disease progression, resulting in a global response of 37%. Similar to the MAVORIC trial, response rates were higher in SS (47.5%) and mostly in peripheral blood compartment (94%) [26].

A phase II study in 2014 enrolled seven Japanese MF patients [27]. Although participants with human immunodeficiency virus (HIV) were excluded from the trials, some clinical cases utilized MOG in these patients without notable safety concerns, suggesting that this infection was not a contraindication for its use [26,27].

The MAVORIC trial, a phase III, controlled study, randomized patients 1:1 to receive either MOG or vorinostat. Most participants were Caucasian, although 37 black patients exhibited a younger mean age at enrollment (53 vs. 66 years) and a higher frequency of earlier stage MF. However, no significant differences were noted in either response or PFS [28]. The intravenous administration of 1 mg/kg of MOG weekly during the initial 28-day period, followed by administration on days 1 and 15 of subsequent cycles until disease progression, was compared to the daily administration of 400 mg of vorinostat in 372 patients with stage IB-IVB relapsed or refractory MF (n = 204) or SS (n = 168). Out of the 186 patients in the vorinostat group, 136 crossed over to the MOG group due to toxicity (27 patients) or disease progression (109 patients). The mean time to response with MOG was 3.3 months. The primary endpoint of the study, median progression-free survival (PFS), was 7.7 and 3.1 months for MOG and vorinostat, respectively, with an overall response rate (ORR) of 28% and 21% in MF and 37% in SS compared, respectively, to 5;7;2% in the comparison arm. Considering the crossover, no difference in overall survival (OS) was observed, as its median survival was not reached after four years of study enrollment [26]. It has to be noted that patients received various lines of treatment (notably, 33 (8.5%) received immunostimulatory agents, 55 (14.8%) received immune-neutral agents, 49 (13.2%) received immunoinhibitory agents, and 13 (3.5%) received HDACis before the clinical trial started).

A notable issue arising from the MAVORIC trial was the difficulty in drawing meaningful conclusions about OS values due to the crossover design and the need for longer-term follow-up. Additionally, cross-study comparisons of CCR expression levels are limited due to variable CCR4 detection methods, while the minimum CCR4 expression level necessary for MOG activity has not been determined. The protocol-specified threshold for positive CCR4 expression was the presence of 10% of CCR4-positive lymphocytes, but unfortunately, this was not based on any predictive correlation [29].

The MAVORIC retrospective study, which is actively enrolling patients, aimed to analyze all patients treated with MOG in Europe based on real-world evidence. A post hoc subgroup analysis of this study based on ORRs by clinical stage revealed higher responses at higher stages (III, IV, IIB, IA/IIA with 26%, 36%, 16%, and 19%, respectively), with compartment-specific ORRs being 42%, 68%, and 17% for skin, blood, and lymph nodes, respectively [30]. Another post hoc analysis of MAVORIC demonstrated that ORR, PFS, and duration of response did not vary with the number of previous treatments [31]. This finding contrasted with in vitro studies using T-cell lymphoma lines treated with vorinostat and romidepsin, which showed a decrease in CCR4 surface and CCR4 mRNA expression [32].

These results aligned with a retrospective analysis of 39 transplant-ineligible patients with untreated aggressive ATLL, where administration of first-line MOG increased the four-year OS probability by 26.3% compared to patients who only received chemotherapy (46.3% vs. 20.6%) [33].

Finally, in a Japanese phase II study involving 29 patients with PTCL, the ORR was 34% [27]. Real-world evidence also demonstrated that MOG improved OS in patients with relapsed/refractory ATLL, particularly those with acute-type ATLL and skin rash [34]. Fujimura et al. [35] presented a case of palmoplantar MF treated with MOG, suggesting that the decrease in CCL22 levels paralleled disease activity and could be considered a response biomarker, associated not only with bexarotene but also with MOG.

Finally, regarding long-term response maintenance when treating patients with MOG, it is important to note that many of them, including those with an initial complete response, might be treatment-resistant. According to some reports, only 11% of patients will have a response lasting at least 12 months, due to the loss of CCR4 expression and genomic alterations in the CCR4 gene [36]. Some authors have proposed different mechanistic explanations, such as detectable or nondetectable genomic events, loss of CCR4 protein expression, and other undetermined mechanisms when preserved CCR4 is observed [4]. Additionally, there are some mutations associated with a higher response rate and improved survival, such as the C-terminal gain-of-function CCR4 [37,38]. However, nowadays these mutations are not routinely studied in laboratory analyses.

## 5. Real-World Results of Mogamulizumab

Since its approval, several studies have demonstrated the real-world efficacy of MOG outside the MAVORIC trial. Most of these studies focus on ATLL, with some cases and case series dedicated to advanced CTCL.

In several instances, isolated cases with positive outcomes have been reported. Fujimura et al. [39] presented a case of folliculotropic MF with intense facial and trunk involvement but no other affected areas. Researchers decided to treat patients with low-intensity radiotherapy, and subsequently, MOG was administered at the standard protocol dose, achieving a complete response lasting a year and a half. The uniqueness of this case lies in the absence of involvement in other areas and the use of MOG to consolidate the response. Biopsies revealed the disappearance of the lymphoid infiltrate of the follicles and a cytotoxic infiltrate around them, suggesting the maintenance of the response derived from the mechanism of action of MOG. The same author also reported successful treatment with this drug of a palmo-plantar MF in 2020 [35], with no other areas involved. It is intriguing that MOG was selected as a first-line treatment in this patient, with authors justifying this decision based on the high expression of CCR4 in both the skin and subcutaneous tissue. They also measured the levels of CCL22, CCL19, and CXCL10 in peripheral blood before and a month after treatment as markers of disease activity, finding a selective decrease in CCL22. This suggested that MOG might also act on this tumor microenvironment ligand of MF, similar to bexarotene, etoposide, or interferons.

Hisamoto et al. [40] published the treatment of an advanced MF with long-term evolution that showed progression during bexarotene treatment (T3N1M0B0), with the appearance of tumors in the larynx and skin extension. The tumor lesions were treated with radiotherapy, bexarotene was maintained, and MOG was initiated after radiotherapy, resulting in a complete response for 24 months. There was no disease extension and no findings on the positron emission tomography–computed tomography (PET-CT) scan. Although bexarotene is not a first-line treatment for advanced MF, they suggested that this combination might be beneficial in some patients.

Later, several retrospective series have also been published. Amagai et al. [41] reported results from a single center involving 11 patients, 8 of whom had stage IIB or higher MF and 2 of whom had unspecified peripheral CTCL, all with positive CCR4 expression. They used the standard MOG protocol with 1 mg/kg per week for four weeks and then every two weeks. In refractory cases, they added 50 mg etoposide three times a week, achieving a complete response of 50% in patients with MF and 33% in patients with unspecified peripheral CTCL. In addition, the time to next treatment delay (TTNT) was 16 weeks. Using this protocol, the authors managed to improve the results in this patient group compared to the MAVORIC trial without observing significant side effects.

Caruso et al. [42] published their experience with MOG in similar number of patients compared to Amagai, that is, 12 patients in various situations in Italy. All of them were refractory to other systemic treatment lines. They reported positive outcomes in four cases of refractory SS to other lines, one case of SS and adenocarcinoma of the pancreas combined with gemcitabine, one case of advanced MF prior to allotransplantation (Allo-HSCT) treatment, one case of SS in combination with electron body bath and photopheresis, one MF with treatment failure to brentuximab with 10% CD30 expression, and cases of refractory advanced MF. The median follow-up of these patients was long, ranging from 150 days to one year, with a good response in all of them. The real-world experience in Italy suggested that MOG can be used in all stages. It is noteworthy that in this pre-transplant case series with a two-month washout period, they did not find GVHD in their patients.

Molloy et al. [43] presented a series of 13 patients compiled from two centers in Spain and the United Kingdom. All of them were affected by SS (two patients) or MF (11 patients) in stages II or higher with failure to more than one classic systemic treatments. A case of ATLL was also included. All of them had previously been treated with polychemotherapy, phototherapy, electron body baths, methotrexate, brentuximab, atezolizumab, and/or resminostat. The patients received between 1 and 26 cycles of MOG treatment. Two patients remained stable, two progressed, and eight responded, one with a complete response and seven with a partial one. Nodal response was assessed in only 10 patients, and in 9 of them, it remained stable. The treatment with MOG was well-tolerated, with the most common adverse effect being of grade II or III in 95% of patients in the form of fatigue, rash, infection, fever, and diarrhea. The patients tolerated the treatment better than in the MAVORIC trial, obtaining a better response, both in the peripheral blood (76.9%) and in the skin (66.7%).

Jouandet et al. [44] published a retrospective series of 24 patients treated with MOG in French hospitals not included in the MAVORIC study, with patients categorized into complete response, partial response, disease stability, and progression. Complete response was defined by the absence of cutaneous lesions and Sezary cells in peripheral blood, partial response by a significant decrease, and stability by persistence. Progression was defined as the extension of lesions on the skin, the appearance of nodes, an increase in Sezary cells, or visceral involvement. Among 21 patients, they found 13 responders and 8 non-responders. Out of the 21 responders, 16 had SS, and 5 had advanced-stage MF. The response rates were 19% for complete response, 38% for partial response, 5% for stability, and 14% for progression, with an overall 24% mortality rate. The PFS was estimated at 22 months, significantly higher than the average estimated in the MAVORIC study. When comparing responders with non-responders, they found no significant differences as predictors of response. However, a tendency towards a lower response to MOG treatment was observed in male patients, when its introduction was delayed, if multiple therapeutic lines were used, and with the amount of affected body surface area. Almost all patients showed side effects to varying degrees, with an average onset at 21 days, on par with the MAVORIC study. This research is particularly interesting because it is the first conducted in real-world practice with a disease-free period of 22 months, clearly superior to other studies such as the one from Japan with 15 months [34] or the seven months of the MAVORIC trial. These patients were in advanced stages with multiple previous treatment lines, without strict selection criteria like MAVORIC, and it was assumed that response rates decreased with each line.

In an insightful study published by Trum et al. [45], it is emphasized that the real-world efficacy of MOG might be underestimated due to the frequent association with cutaneous rash, which does not allow for a proper assessment of the skin response. They studied the presence of MOG-associated rash (MAR) in a series of 24 patients treated with MOG, affecting to 17 of them (14 with SS and 3 with stage IIB or higher MF). The percentage of patients with MAR (68%) was much higher than the one observed in the MAVORIC study. In addition, a relationship was found between the presence of MAR and the treatment response, since while patients with MAR had an overall response rate (ORR) of 88.2%, those without MAR reported an ORR of 28.5%.

Bigger series appeared later on; Beylot-Barry et al. performed a retrospective study in 14 French centers, including 122 patients (median disease duration of 2.5 years), to evaluate the overall response rate [46]. Patients had been administered a median of three systemic CTCL treatments, and most of them were categorized as stage IIB-IVB with blood involvement. MOG demonstrated effectiveness in this population, as ORR reached 58.7% (95%CI: 48.9–68.1), with rates of 69.5% in SS and 46.0% in MF. In accordance with other studies, the most common adverse events were rash and infusion-related reactions. Also, a patient diagnosed with SS died due to MOG-related tumor lysis syndrome.

Finally, another study aimed to enrich the data from the MAVORIC study with real-world evidence from an Australian cohort [47]. As a result, researchers found that the median overall survival was higher in the reweighted MOG treatment arm than in the subgroup of vorinostat [57.2 months (95% CI 44.3-not reached) vs. 40.0 months (95% CI 29.0–81.9)]. Nevertheless, the hazard ratio did not reach statistical significance.

## 6. Safety of Mogamulizumab in Clinical Assays

The main adverse events in the MAVORIC trial were infusion-related reactions (IRRs) (33%), drug rash (24%), diarrhea (23%), and fatigue (23%) [48]. These IRR reactions were primarily limited to early infusions, mostly occurring in the first or second dose, with most being grade I-II, and only 2% of cases reached grade III. A premedication with acetaminophen and diphenhydramine is recommended preceding the first dose of MOG therapy [49].

In terms of autoimmune diseases, their onset ranged from 4.5 to 24 months after initiating MOG, with a mean of 6.8 months. They are less significant than those associated with the anti-PD1 immune checkpoint inhibitor [50] and it might indicate a good prognosis, correlating with long-term complete responses [51]. When added to immunotherapy, MOG does not appear to significantly increase toxicity.

As Pros are so important to consider the patient in the center of the medical approach, a primary analysis of the MAVORIC study, included some of them, such as the Skindex-29, Functional Assessment of Cancer Therapy-General (FACT-G), ItchyQoL, and EQ-5D-3L measurements, which were reported to be increasingly improved in MOG-treated patients at the six-month assessment compared with vorinostat-treated patients [30]. Considering the impaired quality of life in CTCL patients, and the inclusion of many indexes as secondary key points in the MAVORIC trial, a subanalysis focusing on patient-reported outcomes (PROs) showed a correlation of the improvement and benefit of the drug with a higher functional basal impairment [52].

As dermatologists, we have observed and characterized associated skin disorders (32.2%), serious IRRs (4.7%), and infections, mainly cytomegalovirus (CMV) and pneumonia (0.3–0.5%), which have also been reported. No increase in adverse events in patients older than 70 were observed, but an increased risk of GVHD has been observed in younger patients who underwent allo-HSCT. Notably, in a series that included patients treated with MOG before allo-HSCT, an increased risk of severe GVHD was observed, with 7 out of 49 patients dying due to this condition [53]. According to multivariate analysis, poor levels of albumin, and high corrected serum calcium and lactate dehydrogenase (LDH) were associated with a borderline significant poor prognosis [54,55].

A postmarketing all-case surveillance study (Clinical trial registry number: UMIN000025368) conducted on 294 sites in Japan included 597 patients, with 572 included in the safety analysis [53]. Out of all patients, 8.6% of them underwent allo-HSCT after MOG, with a median interval of 36 days (range 6–191). Among these patients, 73.4% and 38.6% reported at least one adverse event and serious adverse event, respectively. Out of all severe adverse effects, in 42 patients, it resulted in death, mostly related to infections (3.1%) and GVHD (1.2%) [53,54,55]. There is also a potential increased risk of acute GVHD if allo-HSCT is performed immediately after MOG bridging treatment [56]. Several additional articles have reported severe GVHD when MOG is used in close proximity to allo-HSCT. Therefore, its use is not recommended in the 50 days before allogeneic stem cell transplantation [57]. This problem can be avoided by allowing enough time to replete Treg cells to prevent an increased incidence of grade II to IV acute GVHD [56]. Some authors have emphasized the need to analyze MOG blood levels and Treg counts before and after allo-HSCT [58].

Finally, regarding long-term response maintenance when treating our patients with MOG, it is important to note that no additional safety concerns have been observed despite some patients developing resistance to treatment [36,37,38].

To compile all the previous information, we can conclude that MOG, based on meta-analysis, has demonstrated clinically meaningful antitumoral activity with an acceptable toxicity profile.

## 7. Mogamulizumab Results with Combined Treatments in ATLL

When facing aggressive lymphomas such as ATLL, there is a significant rationale to combine MOG with other treatments that can stimulate the immune response, such as interferon alpha. Patino et al. [31] reported the results in three patients using this combination [31]. In addition, interleukin 12 (IL-12) also stimulates natural killer (NK) cells to produce interferon (IFN), and topical toll-like receptor agonists induce dendritic cells to produce IFN and IL-12, creating a potent antitumoral response. However, the combination of MOG with a programmed cell death protein 1 (PD1) inhibitor seems to increase the risk of immune-mediated serious adverse events.

A recent systematic review of survival outcomes for relapsed or refractory angioimmunoblastic T-cell lymphoma (AITL) showed that MOG was the most frequently studied treatment regimen, and it can provide longer survival compared with chemotherapy alone. Nine subgroups were treated with MOG, two pre-allo-HSCT, and another one was treated with chemotherapy. The median OS were 2.2–17.6 months for MOG, 3.8–6.2 months for MOG + allo-HSCT, and 4.1–20.3 months for other chemotherapy [59].

Advances in precision medicine with genetic studies on 64 MOG-naïve patients treated for ATLL identified somatic alterations in ATLL cells that influence the clinical outcome of patients treated with MOG. TP53 and CD274 alterations were independently and significantly associated with worse OS, while CCR4 alterations were associated with better OS [60].

By the end of 2022, a patient with relapsed ATLL post-transplant was treated after cytotoxic chemotherapy failure with a single dose of MOG, inducing complete remission for a follow-up of one year with moderate acute GVHD. As reported here and in previous literature, MOG seems to potentiate allogeneic immune antitumor reactions and can be used to treat or prevent relapse without excessive toxicity [61,62,63,64,65,66].

Two different studies comparing mLSG15 addition to MOG to treat aggressive ATLL have been conducted. The first was a phase II study (NCT01173887) evaluating whether the addition of MOG to mLSG15 increases efficacy in patients with newly diagnosed aggressive ATLL, with 29 and 24 patients showing better ORRs in the mLSG15 + MOG arm (86% vs. 75%) [67]. The second one, UMIN000013294, found no differences in survival between the mLSG15 + MOG and mLSG15 groups [67].

Therefore, despite the need for further exploration, the combination of mLSG15 + MOG might be a plausible therapeutic option for newly diagnosed aggressive ATLL patients who are ineligible for allo-HSCT [67]. A retrospective analysis of 77 patients with this condition who were treated with either MOG + EPOCH, MOG + mLSG15, or chemotherapy alone, showed that the addition of MOG to chemotherapy did not result in a survival benefit compared with chemotherapy alone. However, a relationship between the overall survival benefit and the MOG-associated reaction in the skin was observed [68].

## 8. Mogamulizumab Results with Combined Treatments in Cutaneous Lymphomas/Dermatology

Despite the off-label nature of combining MOG with other treatments for cutaneous lymphomas, most reported cases demonstrate a combination of MOG with ultraviolet light (UVB), radiotherapy (localized either low-dose skin electron beam therapy), bexarotene, or even allo-HSCT.

The combination of ultraviolet light B (UVB) with MOG was retrospectively investigated in seven patients with CTCL, revealing photosensitivity reactions. Further phototesting identified the action spectrum in UVB in three cases and in both UVB and ultraviolet light A in one case. Histopathologically, a lichenoid reaction with CD8+ T cells, similar to chronic actinic dermatitis (CAD), was observed to be consistent with other described MOG-associated drug reactions (MAR). The patients in the analysis had MF (four individuals), SS, ATLL, and Epstein–Barr virus T-cell lymphoproliferative disorder (EBV T-LPD) (one patient for each). The minimal erythema dose (MED) decreased to 20–30 mJ/cm^2^, lower than that reported among healthy Japanese subjects (range 90–110 mJ/cm^2^). Therefore, the authors considered MOG-induced photosensitivity as an immune-related adverse event (irAE) virtually identical to CAD [69,70].

A 45-year-old Japanese man with relapsed folliculotropic MF was treated with intensity-modulated radiotherapy (25 Gy) in combination with MOG, achieving complete control of the disease. The authors highlighted that the abscopal effects of MOG followed by radiotherapy were helpful in this case [39].

The use of low-dose total skin electron beam therapy (LD-TSEBT) has a strong biological rationale to treat cutaneous lymphomas, and ongoing clinical phase II and III trials are combining MOG and LD-TSEBT (NCT04256018; NCT04128072). Additionally, using LD-TSEBT (12 Gy) can stimulate immunity by releasing tumor-specific antigens [71,72]. Notably, MOG does not seem to worsen radiation dermatitis, as evidenced in a retrospective study reviewing 46 courses of radiotherapy administered to 15 consecutive patients with ATLL between 2012 and 2017 [73].

Two cases of MOG-resistant MF were successfully treated with additional administration of etoposide. Chemotherapy administration might gather tumor-associated macrophages (TAMs) without increasing C-C motif chemokine 22 (CCL22) and repolarizing TAMs, which could be a therapeutic target for the treatment of MF [74].

Contrary to theoretical concerns, the attenuation of response by decreasing CCR4 expression due to the previous use of HDACi is not supported by the MAVORIC trial, as the crossover from vorinostat had similar results, as do other pieces of evidence [32,75,76]. MOG in combination with extracorporeal photopheresis is also under further testing (NCT04930653).

Regarding the combined therapy of bexarotene and MOG, a published case of a 67-year-old male patient stated that this combination was a successful treatment, in which bexarotene and narrowband UVB were administered before MOG, and radiotherapy was also used in tumoral lesions. This case was also peculiar, as he presented intraoral involvement, which is extremely rare [40,77]. Four additional reported cases showed good tolerability, although half of the patients developed myocardial infarction. Nevertheless, the mechanism of cardiotoxic adverse events is not clear, as the increase in cytokines and TNF, mediators of myocardial infarction and inflammatory factors, might be induced by MOG, leading to thrombosis and vasoconstriction [31,78]. However, real-world evidence indicates that there is not a clinically significant problem in the MOG + bexarotene combination or in using MOG after previous treatment with HDACi [31].

In an article reporting the efficacy of MOG in patients prior to allo-HSCT, it was stated that one individual developed grade IV acute GVHD despite passing more than 200 days between treatment administration and the appearance of the symptoms [56]. Therefore, the performance of blood level testing and Treg counts before and after allo-HCT has been proposed to avoid this adverse event [58]. However, it is important to highlight that despite the 17-day half-life of MOG, Treg counts might remain low for six months or longer [57].

There are no articles combining MOG and interferon, but due to the similarities between MAR rash and irAEs in solid malignancies, the upregulation of interferon-stimulated genes in the skin of patients with MOG-associated adverse events is consistent with the efficacy of interferon alfa in CTCL. These agonists could be used in combination with MOG to enhance its antitumoral immune-induced response [79,80].

## 9. Cutaneous Adverse Events

**Figure 3:**
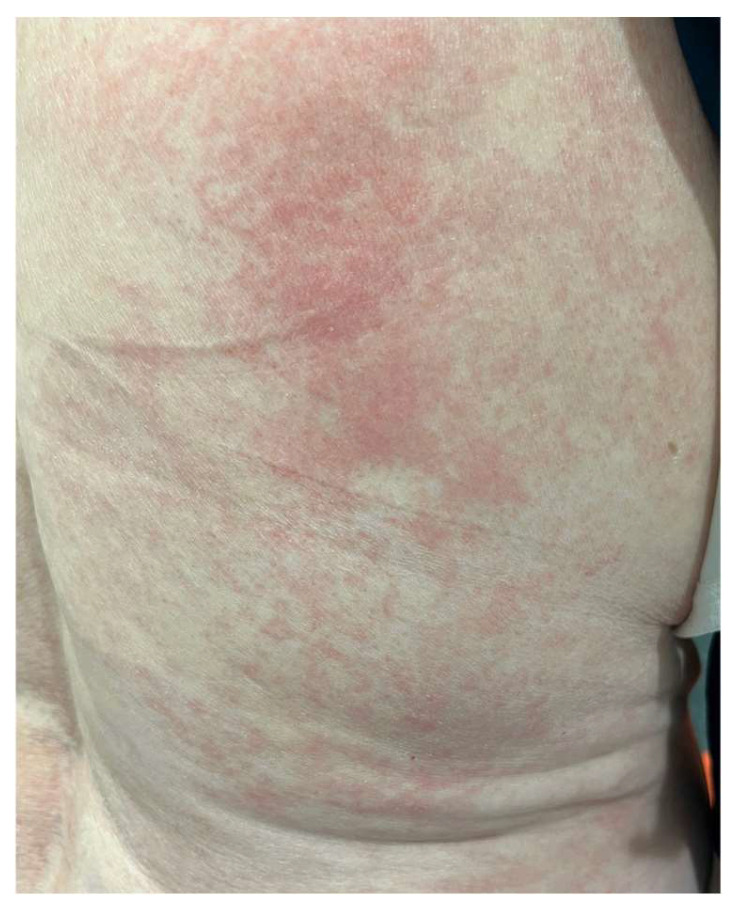
Morbiliform rash in the back of a patient during MOG treatment.

The MAVORIC phase III trial, comparing MOG and vorinostat in previously treated CTCL, reported that skin rash was present in 24% of patients, with photosensitivity, eczematous dermatitis, erythroderma, and neutrophilic eccrine hidradenitis algo being reported. In addition, treatment had to be ceased in up to 7% of patients due to skin toxicity. The most common grade III adverse events included pyrexia and cellulitis, accounting for 4% and 3% of cases, respectively [70] (Figure 3).

Concerning the pathogenesis of MAR, the depletion of CCR4+ Treg cells by MOG is suggested to not only increase antitumor immunity, but also to elicit cutaneous inflammatory responses, as demonstrated in Foxp3 and CCR4 knockout murine models [70]. The blockade of CCR4 leads to impaired Treg global function and an increased inflammatory response against commensal organisms, resulting in a GVHD interface dermatitis [81], or a Th1 cell polarization along with a granulomatous dermatitis [79], which can typically appear in combined patterns [82], as Treg cells’ function is to ensure tissue immune homeostasis and tolerance of commensal organisms both on the skin and in the enteric system [83]. Robust CD8 T-cell proliferation and diversification after MOG in patients with ATLL or CTCL is also associated with MAR appearance [84].

As clinicians, we must consider a skin biopsy in these types of patients when either morbilliform rash, plaques, or any other nontransient cutaneous lesion appears in our patients. It is important to notice that MAR might mimic histopathological features of MF, and we must not stop the treatment directly but closely follow up our patient. We will show how to differentiate both clinical scenarios in the next paragraphs by using mostly IHC as well as the global histopathological pattern.

Expanded histopathological features of MAR include epidermal spongiosis, vacuolar interface change, and epidermal infiltration by small lymphocytes expressing CD8 and cytotoxic markers such as granzyme, perforin, and TIA-1. Additionally, immunohistochemistry revealed a normalized or decreased ratio of CD4:CD8 expression among lymphocytes and a predominance of Th1 over Th2 cells and reduced Treg cells. Histopathologically, the main pattern of MAR combines interface dermatitis and granulomatous dermatitis, with combined epidermal spongiotic or psoriasiform changes. Additional histopathological features mimicking CTCL, such as lymphocyte exocytosis, follicular destruction, and lamellar fibroplasia, can also be observed. MAR is mostly polyclonal, based on high-throughput sequencing of the T-cell receptor [85]. The best way to increase diagnostic accuracy is to compare these lesions with the original lymphoma.

Immunohistochemistry is predominantly useful in distinguishing MAR from MF or SS, as MAR shows predominantly CD8+ T cells, without loss of CD7, and no T-cell receptor (TCR) monoclonality. Clinically, four patterns of MAR have been identified, mimicking folliculotropic MF, MF papules, photoaccentuated dermatitis, or morbilliform reaction [86], as later validated and expanded by Hirotsu et al. [87].

Furthermore, attention should be paid to the fact that granulomatous infiltrates can also involve the bone, and in such cases, it is important to avoid confusion with a recurrence of the lymphoma to prevent overtreatment [88]. MAR can also affect the hair, leading to alopecia [89]. Raval et al. [90] described four patients with MAR-related alopecia, which gradually developed on the parietal and occipital scalp between 10 and 23 months after treatment initiation and showed no hair regrowth after an average of 12 months of follow-up, despite using 12-week courses of high-potency topical steroids. Scalp biopsies showed mixed granulomatous and lichenoid inflammatory infiltrate with eosinophils and overt follicular destructions. Additionally, a case of universal alopecia areata was also described, likely as a consequence of absent Treg cell-mediated inhibition of an autoimmune attack secondary to MOG, which regrew after 13 months with intralesional and topical corticosteroid treatment.

Masson et al. [91] recently published a study on 44 patients (9 MF and 35 SS), with a mean age of 66 years, of whom 32% developed at least one rash during the follow-up period. The mean time to the first rash was 100 days. The previous stage at baseline varied, and the mean number of previous treatment lines ranged from one to nine. Notably, 36% of patients with MAR also presented other associated irAEs, such as alopecia areata, thyroiditis, and hepatitis. Four patients developed a second skin rash after treatment rechallenge. Here, it is important that they demonstrate a longer time to progression in patients with MAR compared to the ones without it. Previously described histopathological patterns were also found, with predominantly intra-epidermal CD8+ T cells in 72% of the cases. Transcriptomic studies showed a distinctive pathophysiology involving CD8 T-cells and CXCL9 and CXCL11 expression, two macrophage-derived chemokines that were upregulated in the skin of MARs, and the key players were macrophage–lymphocyte mediators in the context of CTCL. Another cohort of 72 patients corroborated the longer remission in patients with cutaneous lesions consistent with MAR [92]. This association between cutaneous reaction and good prognosis also occurs in patients treated for ATLL [93].

**Figure 4:**
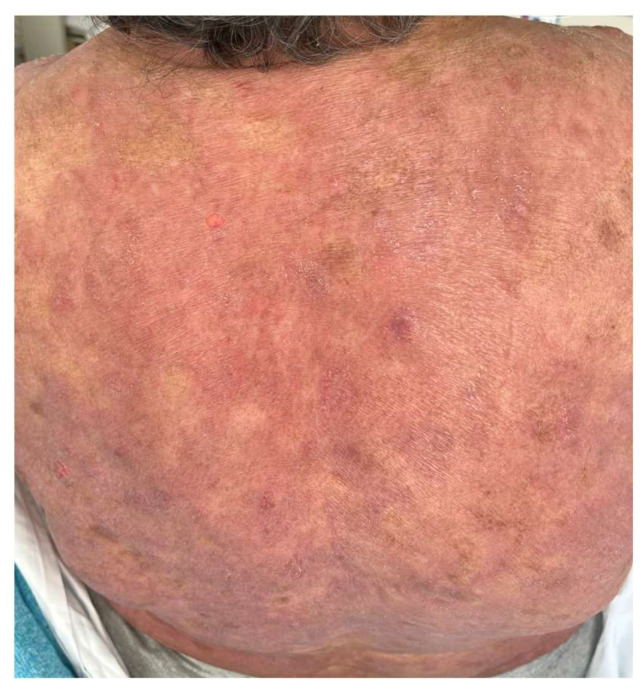
Patient treated with MOG who developed lichenoid reactions that recurred several times during the follow-up.

**Figure 5:**
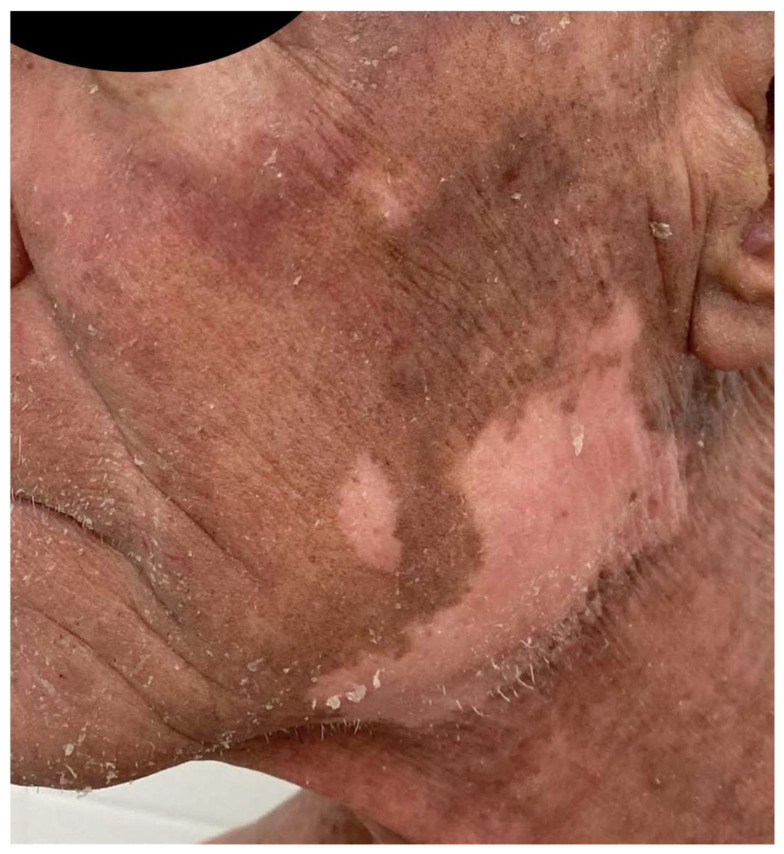
Patient treated with MOG who developed facial vitiligo months after the start of the treatment 2 years ago.

A French cohort of 21 patients reported that 47% of patients developed adverse events, a value comparable to the MAVORIC study, which mostly appeared within the first infusions [44]. Other peculiar cutaneous reactions related to MOG have been published. Trager et al. [94] described mucocutaneous lichenoid reactions and postulated that they might be related to different biological drugs, and could be considered as a class effect, as they can also appear related to rituximab, anti-PD1, anti-CTLA4, or anti-TNF-α therapy. (Figure 4). A few cases of psoriasis relapse have also been observed [95], as well as delayed vitiligo appearance, up to eight months after the initiation of treatment, due to CD8+ T-cell infiltration and destruction of melanocytes, appearing as a good response prognostic factor (Figure 5).

It is important to note that vitiligo lesions have been previously associated with other CTCL treatments, such as interferon or extracorporeal photopheresis [96,97]. There have been cases of MOG-induced Stevens–Johnson syndrome and toxic epidermal necrolysis, which can lead to the death of the patient due to a loss of control of cytotoxic CD8+ T cells secondary to FoxP3 inhibition [98,99,100,101].

Furthermore, a MOG-related radiation recall dermatitis has been reported, although the exact mechanism is still unclear [102,103,104,105]. Finally, eruptive lentiginosis, similar to those reported to be associated with some biological therapies in psoriasis, has been described in one patient [106]. Ecthyma gangrenosum can complicate MOG treatment of SS and is mostly related to the global immunosuppression induced by the disease itself, as CTCL tumoral lymphocytes present a mostly Th2 profile and produce TGF-beta [107]. Other infectious-related problems, such as EBV T-LPD involving the central nervous system (CNS), which develops in severely immunocompromised subjects [108,109,110], mostly with impaired T-cell immunity, have been reported in a patient with AITL with incomplete T-cell reconstitution who received conventional chemotherapy and MOG [111].

Treatment strategies for MARs are currently not standardized, partly due to the involved mechanisms being unknown. Typically, topical steroids are used for grade I-II eruptions, while more severe cases might require administration of systemic steroids or even methotrexate and other systemic therapies [42,44,86,87,88,89,90,91,92,93,94,95,96,97,98,99,100,101,102,103,104,105,106,107,108,109,110,111]. Most grade II or III rashes are usually managed with an oral steroid dose ranging from 0.5 to 1 mg/kg, followed by a short tapering over one or two weeks. If the cutaneous lesions persist, clinicians might consider steroid-sparing treatments such as methotrexate or dupilumab, both of which are acceptable in MF/SS. Primary immunosuppressive agents like cyclosporine should be avoided [112]. Notably, the MAR granulomatous rash in the head and neck, resembling MF, can also be effectively treated with methotrexate at a dosage of 15–20 mg per week or with dupilumab, particularly when larger body surfaces, exceeding 30% of body surface area, are involved [45].

It is important to recognize that systemic therapies for MAR were not permitted in the MAVORIC clinical trial. Based on previous experience, there is no recommendation to discontinue MOG treatment if MAR appears, as it serves as a good prognostic marker [45,113].

## 10. Conclusions

MOG represents a novel targeted therapy that has significantly enhanced the treatment options available for advanced MF and SS. Despite a response rate of 28% in the MAVORIC trial, which is not significantly different from rates observed in other smaller clinical trials such as those involving romidepsin or brentuximab vedotin, MOG provides specific advantages, including a prolonged duration of response and a high response rate within the blood compartment. Additionally, it demonstrates a favorable safety profile, with the presence of irAEs associated with a longer response, attributed to the enhancement of a Th1 antitumoral immune response and modulation of the tumor microenvironment. However, the emergence of resistance after 12–14 months is a primary concern, likely stemming from downregulation or mutation of the CCR4 receptor. Given its mechanism of action, MOG has the potential to form the basis of a wide array of combination therapies that might offer our patients incremental efficacy, an area that warrants further exploration and study.

## 11. Future Directions

Since MOG approval in Europe in 2018, it has been successively included in guidelines for this indication based on data from the MAVORIC study. However, the chronicity of CTCL and the absence of a cure suggest a cautious management of treatment lines and consideration of not introducing toxicity in these heavily treated patients. The relative safety profile of MOG and the real-world experience of its use may allow it to be one of the first options for systemic treatment in the case of therapy failure. We agree with some authors who suggest not delaying the introduction of targeted therapies in CTCL, as, in addition to accumulating toxicity with previous treatments, it could lead to a loss of efficacy.

Real-world efficacy rates of MOG appear to be higher than in the MAVORIC study, and the high frequency of MARs seems to be related to the mechanism of action rather than hypersensitivity to MOG, and dermatologists can manage it properly. Real-world application also suggests that MOG may act through complementary mechanisms associated with its mode of action via immunomodulatory effects on Treg cells. Regarding resistance mechanisms, further studies to understand them and to try to avoid them are warranted.

The introduction of MOG in daily practice will lead to further understanding of its use, and it seems sensible to start directing the treatment of these patients based on molecular biology, skin and blood markers, and flow cytometry.

## Data Availability

Not applicable.
